# Onyxis—the Nail Grown into the Flesh

**Published:** 1843-09

**Authors:** 


*Onyxis—the nail grown into the flesh.*—M. Gerdy does not approve of the avulsion of the nail as a means of curing this painful affection. The nail soon grows again, and with its growth returns the disease. Neither does he approve of the plan of contracting the point of the nail. Matters soon become as bad as ever. Neither is the plan of proceeding, according to which the diseased cushion of parts to the outside of the nail, although superior to the other two, to be greatly commended. The continual pressure upon the pulp of the toe by and by forces a new portion of flesh into contact with the nail, and renewed pain demands renewed interference fro[illegible] the surgeon. "The true and rational method of dealing with [illegible]he affection," says M. Gerdy, "is to remove at a stroke, and obliquely, even beyond the middle of the pulp of the toe, the lateral and inferior parts which rise to suffer pressure from the edge of the nail, and to ulcerate under it." *Lond. and Edin. Monthly Journ. of Med. Sci.*

